# Effects of a Preconditioning Oral Nutritional Supplement on Pig Livers after Warm Ischemia

**DOI:** 10.1155/2012/783479

**Published:** 2012-06-25

**Authors:** Arash Nickkholgh, Zhanqing Li, Xue Yi, Elvira Mohr, Rui Liang, Saulius Mikalauskas, Marie-Luise Gross, Markus Zorn, Steffen Benzing, Heinz Schneider, Markus W. Büchler, Peter Schemmer

**Affiliations:** ^1^Department of General and Transplant Surgery, Ruprecht-Karls University, 69120 Heidelberg, Germany; ^2^Institute of Pathology, Ruprecht-Karls University, 69120 Heidelberg, Germany; ^3^Central Laboratory, Ruprecht-Karls University, 69120 Heidelberg, Germany; ^4^Fresenius Kabi Deutschland GmbH, 61440 Oberursel, Germany; ^5^HealthEcon AG, 4051 Basel, Switzerland

## Abstract

*Background*. Several approaches have been proposed to pharmacologically ameliorate hepatic ischemia/reperfusion injury (IRI). This study was designed to evaluate the effects of a preconditioning oral nutritional supplement (pONS) containing glutamine, antioxidants, and green tea extract on hepatic warm IRI in pigs. *Methods*. pONS (70 g per serving, Fresenius Kabi, Germany) was dissolved in 250 mL tap water and given to pigs 24, 12, and 2 hrs before warm ischemia of the liver. A fourth dose was given 3 hrs after reperfusion. Controls were given the same amount of cellulose with the same volume of water. Two hours after the third dose of pONS, both the portal vein and the hepatic artery were clamped for 40 min. 0.5, 3, 6, and 8 hrs after reperfusion, heart rate (HR), mean arterial pressure (MAP), central venous pressure (CVP), portal venous flow (PVF), hepatic arterial flow (HAF), bile flow, and transaminases were measured. Liver tissue was taken 8 hrs after reperfusion for histology and immunohistochemistry. *Results*. HR, MAP, CVP, HAF, and PVF were comparable between the two groups. pONS significantly increased bile flow 8 hrs after reperfusion. ALT and AST were significantly lower after pONS. Histology showed significantly more severe necrosis and neutrophil infiltration in controls. pONS significantly decreased the index of immunohistochemical expression for TNF-*α*, MPO, and cleaved caspase-3 (*P* < 0.001). *Conclusion*. Administration of pONS before and after tissue damage protects the liver from warm IRI via mechanisms including decreasing oxidative stress, lipid peroxidation, apoptosis, and necrosis.

## 1. Introduction

 During liver surgery, the inflow occlusion maneuver to prevent blood loss as well as the liver manipulation itself have been shown to induce a cascade of molecular events, referred to as ischemia-reperfusion injury (IRI). IRI leads to the activation of Kupffer cells (KCs), the release of reactive oxygen species (ROS) and proinflammatory cytokines, microcirculatory disturbances, and eventually liver dysfunction and failure [1–10]. Different strategies have been proposed to prevent or ameliorate IRI. Among others, pharmacological preconditioning has been shown to be effective via mechanisms including, but not limited to, the direct neutralization of ROS, upregulation of anti-inflammatory, and downregulation of proinflammatory signaling pathways [11–27].

During IRI, intestinal endotoxins (LPS) leak through the altered gut membrane into the portal circulation and enhance the phagocytosis in hepatic KCs [28–35]. This interrelation between intestinal LPS and hepatic KCs makes the gastrointestinal tract an attractive target for the pharmacological preconditioning strategies against hepatic IRI. We hypothesized that an oral pharmacological preconditioning supplement, tailored not only to exert direct ROS-scavenging activity but also to stabilize the gut epithelium during IRI, would tackle the warm hepatic IRI in a porcine model. To the best of our knowledge, this work is the first report of an oral pharmacological preconditioning against hepatic IRI in a larger animal model.

## 2. Materials and Methods

### 2.1. Animal Care

German landrace pigs (32.3 ± 0.9 kg) were given access to standard laboratory chow (ssniff R/M-H, ssniff Spezialdiäten, Soest, Germany) and tap water *ad libitum* before experiments. All experimental procedures were reviewed and approved by the responsible authority (Regierungspräsidium Karlsruhe, Baden-Württemberg, Germany) according to the animal welfare legislation (§ 8 Abs. 1 Tierschutzgesetz (TierSchG) dated 18 May, 2006 (BGBI. I S. 1206)) and were performed according to institutional guidelines at the Ruprecht-Karls University of Heidelberg.

### 2.2. Experimental Procedure

**Figure 1:**
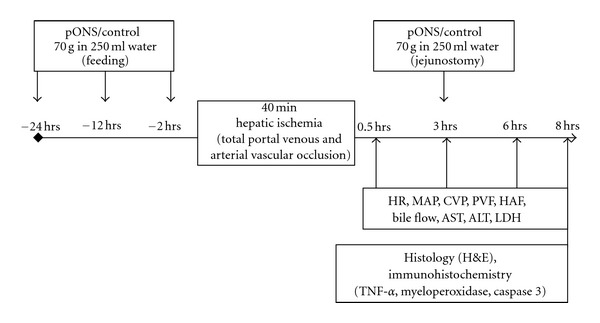
Experimental design. pONS (70 g in 250 mL tap water) was given to overnight-fasted German Landrace pigs 24, 12, and 2 hrs before warm ischemia of the liver. A fourth dose was given 3 hrs after reperfusion. Controls were given the same amount of cellulose. Two hrs after administration of the third dose, both the portal vein and the hepatic artery were clamped for 40 min. After reperfusion, hemodynamic parameters, bile production, and transaminases were measured serially. Liver tissue was taken 8 hrs after reperfusion for histology (H&E) and immunohistochemistry (TNF-a, myeloperoxidasby the Mann-Whitney rank sume, cleaved caspase-3) as described in Materials and Methods. pONS: preconditioning oral nutritional supplement; hrs: hours; min: minutes; PR: pulse rate; MAP: mean arterial pressure; CVP: central venous pressure; PVF: portal venous flow; HAF: hepatic artery flow; AST: aspartate aminotransferase; ALT: alanine aminotransferase.

Pigs underwent general anesthesia. After premedication with Azaperone (Stresnil, Janssen-Cilag Pharma, Wien, Austria, 1-2 mg/kg, i.m.) and midazolamhydrochloride (Dormicum 15** **mg/3 mL, Roche, Grenzach-Wyhlen, Germany, 0.5–0.7 mg/kg, i.m.), anesthesia was induced with Esketaminhydrochloride (KETANEST S 25 mg/mL, Parke-Davis, Berlin, Germany, 10 mg/kg i.v.) and midazolam hydrochloride (1–1.4 mg/kg i.v.). After endotracheal intubation, animals were ventilated with a mixture of 1.5–2.0 L/min oxygen, 0.5–1.0 L/min air, and 0.75%–1.5% isoflurane (Isofluran-Baxter, Baxter, Unterschleißheim, Germany, semiopen ventilation). For analgesia, Piritramide (Dipidolor, Janssen-Cilag, Neuss, Germany, 3.75 mg/h intravenously) was administered. Body temperature was maintained using warming blankets (WarmTouch, Maleinckrodt Medical GmbH, Hennet/Sieg, Germany) and monitored by continuous rectal temperature probes. Systemic hemodynamic parameters, including mean arterial pressure (MAP) and central venous pressure (CVP), were measured continuously (Stetham Transducer, Hellige Monitoring, Freiburg, Germany) by indwelling polypropylene catheters (Braun, Melsungen, Germany) in the common carotid artery and internal jugular vein, respectively. Heart rate (HR) was monitored by body surface electrocardiogram recordings. Experimental groups were given a preconditioning oral nutritional supplement (pONS, 70 g per serving, Fresenius Kabi, Germany) containing glutamine, green tea extract (the resource, method of extraction, and composition of green tea extract has been published elsewhere [36]), vitamin C, vitamin E, beta carotene, selenium, zinc, and carbohydrates (1 sachet = 70 g) (Table 1) dissolved in 250 mL tap water 24 hrs (p.o.) and 12 hrs (p.o.) before the operation. The animals were then fasted overnight. On the day of operation and after performing a midline laparotomy, a third dose of pONS was applied via a jejunostomy tube. The portal vein and common hepatic artery were then mobilized and encircled by elastic bands. Two hrs after the administration of the third dose of pONS, the portal vein and the common hepatic artery were closed with Yasargil clamps (Aesculap, Tübingen, Germany) for 40 min to induce warm ischemia. Common bile duct was cannulated to collect bile continuously. After 40 min, the liver was reperfused by removing the clamps. A fourth dose of pONS was given 3 hrs after reperfusion. Controls were given the same amount of cellulose with the same volume of water. Serial blood samples were drawn and spun at 0.5, 3, 6, and 8 hrs after reperfusion and serum samples were kept at −20°C for the analysis of transaminases (aspartate aminotransferase (AST) and alanine aminotransferase (ALT)) serum concentrations with standard enzymatic methods [37]. The changes in bile production during each time interval were documented and the amount of the newly produced bile was plotted at the end of each time interval to assess the bile flow rate over time. Liver tissue was taken 8 hrs after reperfusion for histology (hematoxylin and eosin (H&E) staining) and immunohistochemistry (TNF-*α*, myeloperoxidase, cleaved caspase-3). Hemodynamic parameters (HR, MAP, CVP, PVF, HAF) were continuously monitored throughout the experiments; ultrasonic probes (Transsonic System Inc, New York, NY, USA) were used for the measurement of portal venous flow (PVF) and hepatic arterial flow (HAF). The experimental design is outlined in Figure 1. After the completion of experimental procedures 8 hrs after reperfusion, animals were sacrificed in deep anesthesia through the intravenous application of a high dose of potassium chloride. 

### 2.3. Histology

Liver samples were fixed by perfusion with 5% paraformaldehyde in Krebs-Henseleit bicarbonate buffer (118 mmol/L NaCl, 25 mmol/L NaHCO3, 1.2 mmol/L KH2PO4, 1.2 mmol/L MgSO4, 4.7 mmol/L KCl, and 1.3 mmol/L CaCl2) at pH 7.6, embedded in paraffin, and processed for light microscopy (H&E) 8 hrs after warm ischemia. In order to assess the histomorphological changes, 40 areas of 0.15 mm² were evaluated per slide using a point-counting method as described previously [38]: grade 0, minimal or no evidence of injury; grade 1, mild injury, including cytoplasmic vacuolation and focal nuclear pyknosis; grade 2, moderate to severe injury with extensive nuclear pyknosis, cytoplasmic hypereosinophilia, and loss of intercellular borders; grade 3, severe necrosis with disintegration of hepatic cords, hemorrhage, and neutrophil infiltration. To describe leukocyte infiltration into the hepatic tissue, a scale from 1 to 4 was used: grade 1, <10 leukocytes/field (focal infiltration); grade 2, 10–20 (mild infiltration); grade 3, 21–50; grade 4, >50 leukocytes/field.

### 2.4. Immunohistochemistry

Paraffin sections from liver tissue obtained 8 hrs after reperfusion were deparaffinized in xylene and rehydrated with graded ethanol. Antigen retrieval was performed via microwave pretreatment in EDTA buffer (pH 9.0) three times for 5 min. The specimens were then cooled and treated with 30% hydrogen peroxidase (H_2_O_2_) in phosphate-buffered saline (PBS)—final H_2_O_2_ concentration: 1%—to block endogenous peroxidases. Nonspecific antibody binding was blocked by normal rabbit serum. Sections were incubated with rabbit polyclonal anti-mouse tumor necrosis factor-alpha (TNF-*α*) antibody (Biosource Europe, Nivelles, Belgium) at the dilution of 1 : 500, and rabbit polyclonal anti-cleaved caspase-3 antibody (DCS, Hamburg, Germany) at a 1 : 200 dilution. After incubation, secondary biotinylated polyclonal rabbit anti-mouse immunoglobulin (Dako, Hamburg, Germany) was applied at a dilution of 1 : 200 for 1 hr followed by streptavidin-biotin complex. For myeloperoxidase (MPO) immunohistochemistry analysis, the sections were pretreated with proteinase K (1 : 40 dilution) and then blocked with bovine serum albumin. They were then incubated with the primary antibody, a polyclonal rabbit anti-MPO antibody (Dako, Carpinteria, CA, USA) at a dilution of 1 : 200, for 60 min at room temperature. A biotinylated swine anti-rabbit antibody (diluted 1 : 300) was used as the secondary antibody.

Positive cells for immunohistochemistry were counted in 10 microscopic fields per slide and slides were evaluated with a semiquantitative technique, relating the score of 0 to 4 points to the fraction of stained cells: scale 0, 0% cells; 1, <5% cells; 2, 5%–20% cells; 3, >20%–40% cells; 4, >40% positive cells as described elsewhere [12].

### 2.5. Statistics

Mean values ± SEM were compared using one-way ANOVA with the Students-Newman-Keuls post hoc test for the analysis of differences in hemodynamic values, vascular flow measurements, bile production, and transaminases. Differences in histological grading of injury as well as in immunohistochemical staining were tested by the Mann-Whitney rank sum test. *P* < 0.05 was selected prior to the investigation as the criterion for significance of differences between groups.

## 3. Results

### 3.1. General and Hemodynamic Data

Hematocrit, body weight, and temperature were not different between control and pONS groups (*n* = 6 in each group) (Table 2). Continuous postperfusion monitoring of the hemodynamic parameters (HR, MAP, CVP, PVF, HAF) also showed no significant differences between the two groups (Table 2). 

### 3.2. Liver Injury and Bile Production

**Figure 2:**
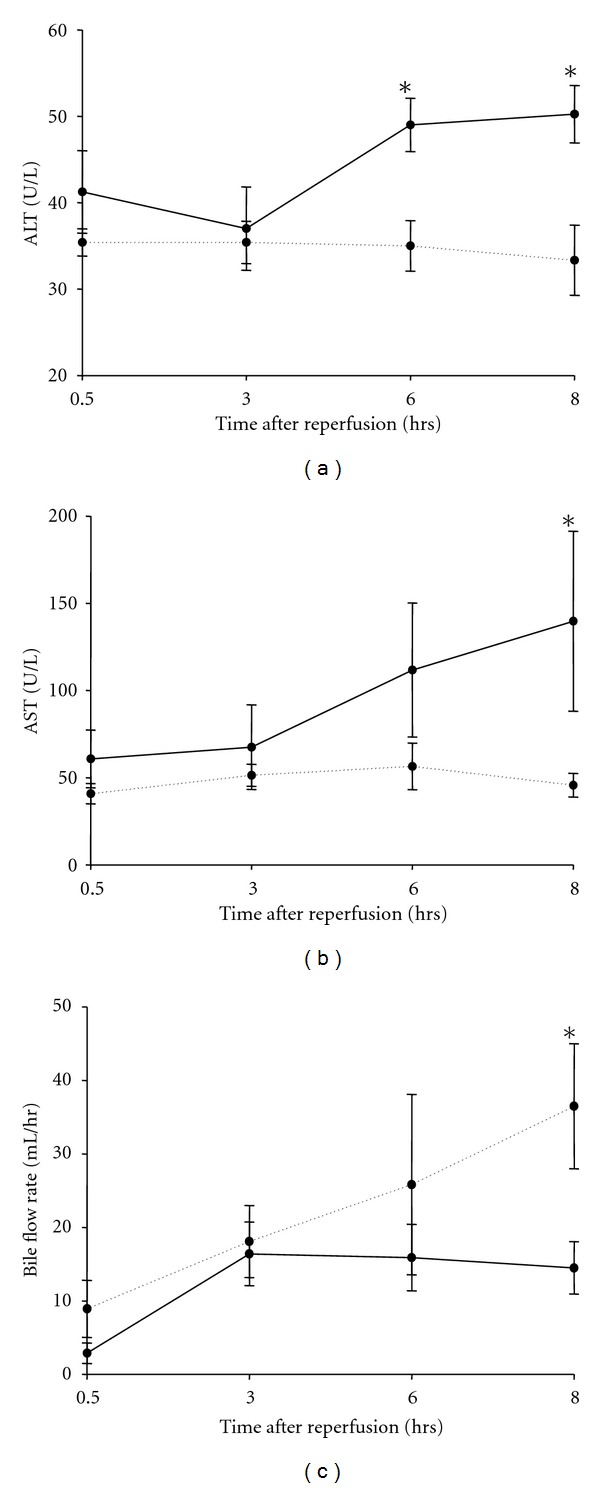
Effects of pONS on serum transaminases and bile production after reperfusion. ((a), (b)) Serial measurement of transaminases was performed after reperfusion as described in Materials and Methods. (c) Bile flow rate (mL/hr) has been depicted over time after reperfusion. Values are mean ± SEM (*P* < 0.05 by one-way ANOVA with Students-Newman-Keuls post hoc test, *n* = 6 per group); *P < 0.05 for comparison to controls; AST: aspartate aminotransferase; ALT: alanine aminotransferase; pONS: preconditioning oral nutritional supplement.

**Figure 3:**
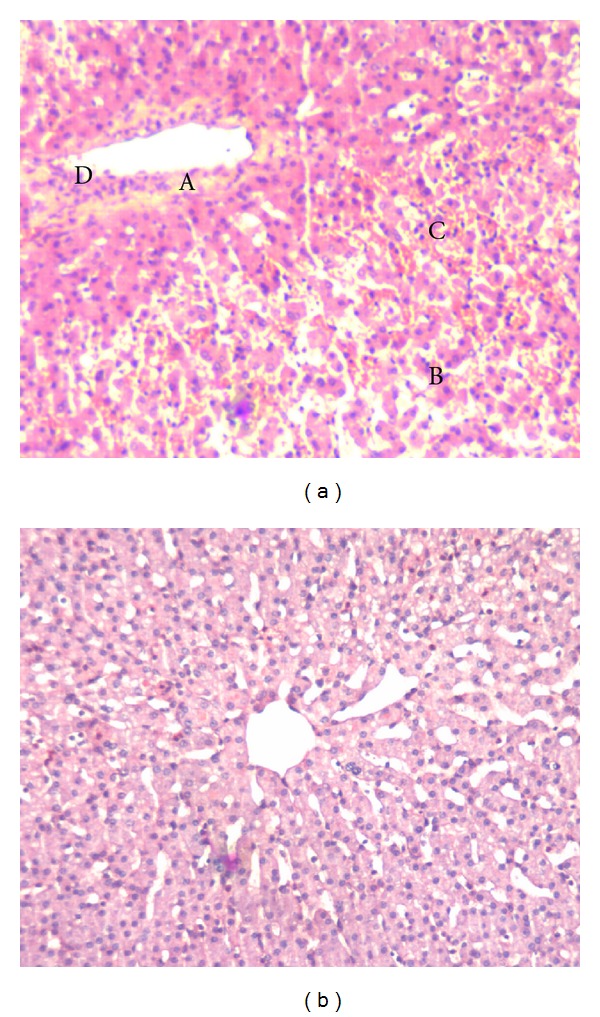
Liver injury eight hours after reperfusion. Liver tissue was taken 8 hrs after reperfusion and processed for light microscopy by H&E staining. (a), control; (b), pONS; controls displayed severe focal necrosis (A) with disintegration of hepatic cords (B), hemorrhage (C), and neutrophil infiltration (D) 8 hrs after reperfusion; this effect was significantly blunted by pONS. Pictures depict typical pattern of pathology; pONS: preconditioning oral nutritional supplement.

While serum ALT increased in controls after warm ischemia/reperfusion to the liver, pONS prevented this effect; the difference between the two groups started to be significant 6 hours after reperfusion (49 ± 3 U/L in controls versus 35 ± 3 U/L in pONS; *P* = 0.01). This difference continued to exist until the end of experiments, 8 hrs after perfusion (50 ± 3 U/L in controls versus 33 ± 4 U/L in pONS; *P* = 0.02). pONS had the same effect on serum AST levels after reperfusion. The difference between the groups was significant 8 hrs after reperfusion (140 ± 52 U/L in controls versus 46 ± 7 U/L in pONS; *P* = 0.01) (Figure 2). There was significantly more severe necrosis with disintegration of hepatic cords, hemorrhage, and neutrophil infiltration (the median grade for necrosis and leukocyte infiltration were 3 and 4, resp.) in control tissue taken 8 hrs after reperfusion. pONS decreased the severity of the above-mentioned histomorphological changes in the liver (the median grade of necrosis and leukocyte infiltration of 1; *P* < 0.001) (Figure 3). Bile flow rate (mL/hr) was significantly higher in the pONS group 8 hrs after reperfusion (Figure 2). 

### 3.3. Immunohistochemistry

**Figure 4:**
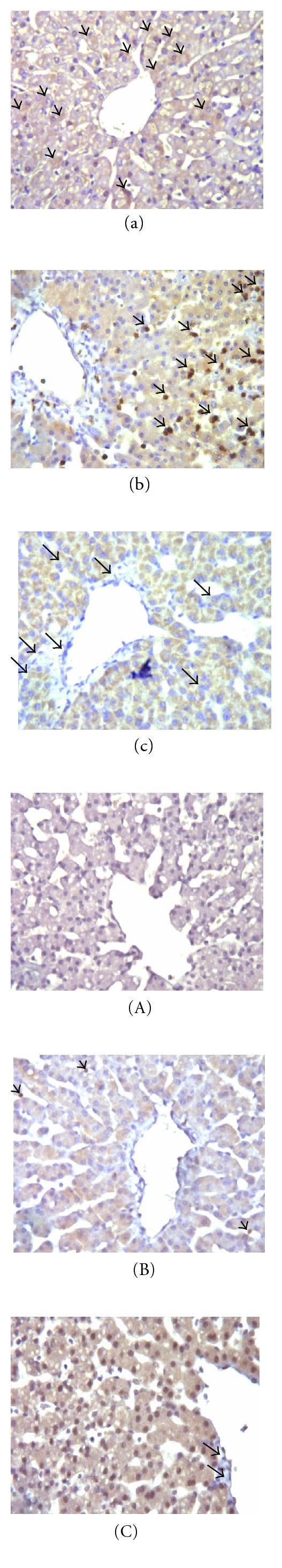
Immunohistochemistry for TNF-*α* ((a), (A)), MPO ((b), (B)), and cleaved Caspase-3 ((c), (C)). Conditions as described in Materials and Methods. Eight hrs after reperfusion, tissue was collected and processed for immunohistochemical analysis with light microscopy. The intensity of TNF-*α* expression (brown staining, black arrows), MPO expression (brown staining, black arrows), and cleaved Caspase-3 (blue halo, black arrows) was significantly higher in controls ((a), (b), (c), resp.) compared to their pONS counterparts ((A), (B), (C), resp.). Pictures depict typical pattern of staining (original magnification: ×200). TNF-*α*: tumor necrosis factor-alpha; MPO: myeloperoxidase; pONS: preconditioning oral nutritional supplement.

The immunohistochemical analysis of sections obtained 8 hrs after reperfusion indicated positive staining for TNF-*α*, MPO, and cleaved Caspase-3. pONS reduced the number of positively stained hepatocytes against all of three above enzymes (Figure 4). The quantitative assessment of immunohistochemical findings is presented in Table 3.

## 4. Discussion

The manipulation of the liver during hepatic surgery activates Kupffer cells, which leads to the release of proinflammatory cytokines, including tumor necrosis factor-alpha (TNF-*α*) and interlukin-1 (IL-1) as well as free radicals, thus initiating an inflammatory cascade [2–7]. This phenomenon is usually further complicated through the application of the Pringle maneuver (hepatic inflow occlusion) in an attempt to prevent blood loss during the hepatic transection [39]. The cumulative effect induces warm IRI to the liver, which results, depending on the duration of ischemia, in microcirculatory disturbances, liver cell damage, and—in severe cases—liver failure [40]. Several approaches have been proposed to pharmacologically tackle hepatic IRI; none, however, has found its way into clinical routine yet.

To prevent IRI, it is important to neutralize reactive oxygen and nitrogen species, either by administering radical scavengers or by enhancing the capacity of endogenous redox defense systems [41]. A vast variety of dietary constituents can exert radical scavenging effects in vivo. Among all, hydrophilic ascorbic acid (vitamin C) and lipophilic *α*-tocopherol (vitamin E) are the important components of the human antioxidant system [42]. Carotenoids, the principal dietary source of vitamin A in humans [43], polyphenolic compounds in green tea extract [36, 44], selenium [45], and zinc [46] all exert antioxidant action; a synergism among the different antioxidants as part of an antioxidant network has been shown [47–49]. 

Large amounts of Gram-negative bacteria and endotoxins (LPS) are normally present in the intestines. A reduction in splanchnic blood flow and ischemia damages the intestinal wall and changes the permeability of the gut membrane, leading to excessive leakage of LPS and bacterial translocation into the portal circulation. It has been shown that LPS can activate KCs directly [50]. Scavenger receptors, including the scavenger receptor cystein-rich (SRCR) superfamily members that are expressed on KCs, are involved in the bactericidal action by binding and endocytosis of endotoxin [51, 52]. The CD11/CD18 receptor of KCs, the pattern recognition receptors (PRRs) CD14, and the Toll-like receptor 4 (TLR4) in combination with the adaptor protein MD2 are reported to be essentially involved in the LPS-associated KC activation [50, 53]. This may reflect an evolutionary adaptation by KCs to their local hepatic environment and strategic anatomic position in the portal circuit, which is optimal for the removal of endotoxin and, thus, for the protection of the host [54]. Glutamine has been shown to have a positive impact on the intestinal barrier by reducing permeability and bacterial translocation and preserving mucosal integrity [55, 56]. It can, therefore, prevent Kupffer cell activation and results in a more favorable outcome [57]. 

However, we did not measure blood LPS levels or histology of intestine, which might have further documented the protective effects of pONS on intestine.

In many models of liver injury, TNF-*α* levels are elevated and correlate with injury; the inhibition of TNF-*α* activity can attenuate liver injury, protect hepatic morphology, and decrease mortality [54]. MPO has been shown to be largely responsible for the neutrophil-induced parenchymal cell killing [58]. Released from the neutrophil's azurophilic granules, MPO can generate hypochlorous acid, a diffusible oxidant and chlorinating agent that gives rise to other toxic species, such as chloramines [59]. Apoptotic cell death can trigger neutrophil transmigration, severely aggravating apoptotic cell injury; caspase inhibitors can have a significant overall protective effect on hepatic IRI [60]. In the present study, we have shown that the oral administration of consecutive doses of a preconditioning supplement in experimental pigs significantly reduced the transaminases compared to controls after hepatic warm IRI. Furthermore, pONS resulted in significantly milder histological changes as well as a significant increase in bile production. The milder histological changes as well as improved sinusoidal bile production after pONS represents reduced postreperfusion injury. This has been further proved by the immunohistochemical analysis of the tissues obtained 8 hrs postreperfusion; pONS reduced the expression of TNF-*α*, MPO, and cleaved Caspase-3. pONS most likely exerts these protective effects via different mechanisms including direct antioxidative effects of its various antioxidant constituents including vitamin C, vitamin E, *β*-carotene, polyphenolic compounds in green tea extract, selenium and zinc. Furthermore, pONS most likely exerts an inhibitory effect on LPS-associated KC activation through glutamine. 

To the best of our knowledge, this work is the first report of an oral pharmacological preconditioning against hepatic IRI in a larger animal model. The application of an oral nutritional substance in pigs is safe, reproducible, and well-deals with the current obstacles faced within the context of hepatic surgery and warm IRI. Tailoring such clinically-oriented experiments may finally help improve bench-to-bedside preconditioning protocols.

## Figures and Tables

**Table 1:** Composition of the pONS.

Component	Dry weight per sachet (g)
Glutamine	15
Antioxidants	
Green tea extract	1
Vitamin C	0.75
Vitamin E	0.25
ß-carotene	0.005
Selenium	0.00015
Zinc	0.01
Carbohydrates	50

1 sachet of pONS was dissolved in 250 mL tap water before use. Conditions as mentioned in Materials and Methods. pONS: preconditioning oral nutritional supplement.

**Table 2:** Basic parameters.

	Control (*n* = 6)	pONS (*n* = 6)	*P*
Body weight (kg)	33.1 ± 1.7	31.4 ± 0.8	0.4
Temperature (^°^C)	36.9 ± 0.2	36.8 ± 0.2	0.7
Respiratory rate (/min)	12 ± 1	12 ± 1	0.9
Hct (%)	30 ± 0.9	33 ± 1.7	0.1
HR (/min)	177 ± 10	185 ± 9	0.6
MAP (mmHg)	90 ± 4.1	91 ± 3.7	0.9
CVP (mmHg)	15 ± 1.7	14.8 ± 0.5	0.9
PVF (L/min)	1.4 ± 0.6	1.9 ± 0.2	0.3
HAF (dL/min)	114 ± 22	117 ± 12	0.9

Table shows basic parameters (body weight, temperature, and respiratory rate) as well as postperfusion data for hematocrit (Hct), heart rate (HR), mean arterial pressure (MAP), central venous pressure (CVP), portal venous flow (PVF), and hepatic arterial flow (HAF). Values are mean ± SEM.

**Table 3:** Quantitative assessment of immunohistochemical findings.

Expression	Control (*n *= 6)				pONS (*n* = 6)				* P*
	*n*	median	25%	75%	*n*	median	25%	75%
TNF-*α*	84	2	2	3	102	1	1	1	<0.001
MPO	79	4	3	4	99	2	2	3	<0.001
Caspase-3	81	4	3	4	100	2	1	2	<0.001

Conditions as described in Materials and Methods; *n* = 6 in each group; median values of indices for immunohistochemical expression of TNF-*α*, MPO, and cleaved caspase-3 with interquartile range 8 hrs after warm ischemia/reperfusion have been compared with Mann-Whitney rank sum. TNF-*α*: tumor necrosis factor-alpha; MPO: myeloperoxidase; pONS: preconditioning oral nutritional supplement.
